# Hepatic metastasis surveillance in uveal melanoma: a retrospective cohort study from a UK tertiary centre (2006–2022)

**DOI:** 10.1038/s41416-026-03445-7

**Published:** 2026-04-16

**Authors:** Benjamin Rea, Mahan Salehi, Ahmed Maiter, Lorraine Ochieng, Sarah Lanham, Joe Kang, Sharabh Sinha, Jenna Hawthorn, Amarit Gill, Christopher Johns, Michael J. Campbell, Umiya Harley, Hibba Quhill, Sachin Salvi

**Affiliations:** 1 Department of Radiology, Sheffield Teaching Hospitals NHS Foundation Trust, Sheffield, UK; 2 School of Medicine and Population Health, University of Sheffield, Sheffield, UK; 3 NIHR Sheffield Biomedical Research Centre, Sheffield, UK; 4 Department of Ocular Oncology, Sheffield Teaching Hospitals NHS Foundation Trust, Sheffield, UK

**Keywords:** Eye cancer, Uveal diseases

## Abstract

**Background:**

Liver surveillance imaging is essential for detecting early asymptomatic metastases in uveal melanoma, which predominantly involves the liver. Early detection may improve treatment opportunities, but variability in imaging protocols and a lack of consensus on surveillance duration present challenges. This study aimed to evaluate our systemic surveillance protocol, optimise pathways, and assess risk factors for metastasis.

**Methods:**

We retrospectively analysed patients diagnosed with uveal melanoma between 2006 and 2021 who underwent hepatic imaging surveillance at Sheffield Teaching Hospitals NHS Foundation Trust. Demographics, tumour characteristics, treatments, disease status, and survival outcomes were collected.

**Results:**

Among 1086 patients (45% female, 79% White; median age 68 years), 315 (29%) developed metastases, with 293 (93%) detected within five years of ocular treatment. The number needed to scan (NNS) increased substantially after five years, indicating reduced detection efficiency. Higher T stage and ciliary body involvement were significantly associated with increased metastatic risk (*P* < 0.01).

**Conclusions:**

Most metastases from uveal melanoma occur within five years of treatment. Personalised, risk-based surveillance strategies considering tumour stage and location may improve efficiency and optimise healthcare resource use.

## Background

In the UK, between 700 to 800 individuals are diagnosed with uveal melanoma (UM) each year [1]. Almost 50% of patients develop metastatic disease, which most commonly involves the liver (93%), lungs (24%), and bones (16%) [2, 3]. The median time from ocular treatment to liver metastasis is 27 months (IQR, 13–46) [4]. Metastatic disease is associated with a poor prognosis, with a median survival of 14–28 months, with only 20% surviving one year or more [2, 5, 6].

Early detection of metastases (i.e., before the onset of symptoms) enhances any opportunities for treatment, identifies patients for possible clinical trial inclusion, and enables earlier palliative care, if needed [7]. Ultrasound is widely used because it offers high spatial resolution enabling identification of small lesions, is quick, inexpensive and widely available. However, it is operator dependent, and sensitivity may be limited in patients with a high BMI or when bowel gas interferes with imaging [8]. Due to these patient factors, it may miss smaller lesions with a quoted specificity of 85% [8]. At our centre, good sonographer expertise and same-day reporting enhance its value, especially when paired with same-day ophthalmic assessment. MRI has a reported sensitivity of 95% and is often used to characterise lesions identified on ultrasound, but this sensitivity decreases with sub centimetre metastases and significantly in metastases <5 mm [9]. A percutaneous biopsy can be obtained if confirmatory histopathology is required [5, 10, 11]. 18-FDG PET is an alternative modality with high sensitivity, but it is not utilised in our centre as the high radiation exposure that would result from repeated examinations limits its applicability as a surveillance tool [12].

The approach to liver surveillance imaging is highly variable both internationally and within the UK, with no established consensus on the optimal imaging technique, frequency or duration due to lack of evidence within the literature [13]. The European Society for Medical Oncology (ESMO) has not established specific surveillance guidelines for uveal melanoma [14]. In the United States (USA) The National Comprehensive Cancer Network (NCCN) provides guidelines for uveal melanoma surveillance utilising risk stratification based on the tumour size, location, histological features, genetic factors and liver function blood test [15]. Intensive imaging is reserved for patients with high-risk features, typically every 3–6 months for the first five years [15]. In the UK, Melanoma Focus recommended a total of 10 years surveillance with six monthly intervals in the first 5 years and annually thereafter [13]. However, surveillance has not been recommended for patients who have small tumours with favourable genetic markers [13]. In Scotland, patients with uveal melanoma are stratified into high or low/medium risk, with high-risk patients undergoing one liver MRI examination every 6 months, for 10 years [16].

Sheffield Teaching Hospitals NHS Foundation Trust (STH) is one of the four National Centres of Excellence for uveal melanoma in the UK [17]. Similar to the Melanoma Focus guidelines, patients undergo hepatic surveillance every six months for the first five years using ultrasound. This is then followed up by annual ultrasound surveillance for up to ten years. Patients with lesions detected on ultrasound, undergo MRI to characterise the lesion, and then undergo temporaneous MRI surveillance. If the patient remains free of metastatic disease on subsequent MR imaging, they preferably return to US surveillance. However, in practice patients may remain on MRI surveillance going forward, reflecting the complexity of factoring in individual clinician and patient preferences into surveillance pathways. All patients within the service currently undergo the same surveillance strategy, irrespective of whether they have a high or low risk primary tumour.

At our centre, the cost of an MRI scan for uveal melanoma surveillance is approximately £150, almost four times the cost of an abdominal ultrasound scan, and the current waiting times for urgent and routine MRI scans are 10 or 16 weeks, respectively. Therefore, a ten-year MRI-focused surveillance programme places significant demands on medical imaging and financial resources [18].

The aim of this study was to evaluate the frequency and timing of liver metastases in patients with uveal melanoma undergoing surveillance imaging in Sheffield and assess the appropriateness of current practice. It also aimed to examine trends in detection efficiency over time and explore clinical factors associated with metastatic risk, including tumour location and T-stage.

## Methods

### Ethics approval and consent to participate

All methods were performed in accordance with the relevant guidelines and regulations. This single-centre retrospective study was approved by the local clinical research office, the local research ethics committee (Sheffield 3D Lab; reference 17/YH/0142), and the information governance team. As this was a retrospective study using non-identifiable data, the requirement for informed consent was waived by the clinical research office and ethics committee.

### Patient selection

Patients were identified retrospectively by searching the Sheffield Ocular Oncology Service database. They were included if more than 16 years old, diagnosed with uveal melanoma between January 2006 and October 2021 and if they underwent hepatic surveillance with ultrasound or MRI at our hospital. Patients were excluded if no hepatic imaging was performed at our hospital after the initial staging scan at STH, or if they had synchronous (cancers diagnosed within 6 months of each other) or metachronous (cancers diagnosed more than 6 months apart) cancers with liver metastases without histological confirmation of the metastatic origin of the liver lesions, or if TNM data was not available. Patients were not pre-selected based on their primary characteristics, including the risk profile of the tumour.

### Data collection

Data was copied to a Microsoft Excel spreadsheet from a combination of the STH CRIS (Computerised Radiology Information System) and PACS (Picture archiving and communication system). For each eligible patient, the following variables were recorded: demographic information (including current age, sex, and self-reported ethnicity); details of initial diagnosis (tumour subtype, TNM staging, and primary treatment); disease status (disease-free or metastatic) and date of death; the date and available findings of the most recent liver surveillance imaging (ultrasound or MRI), noting that no second image interpretation was performed; and the date of the initial documented diagnosis of liver metastasis. With patients who had initial surveillance imaging at STH, with subsequent surveillance imaging at their local centre, the date and report of the latest STH imaging were documented.

Where necessary BR acted as an arbitrator to resolve any discrepancies or conflicts which had been flagged during data collection. In cases of missing data, patients’ electronic health records and letters were reviewed to fill in any gaps. For patients outside of our integrated care system, missing data was collected by contacting their local General Practitioner.

### Data categorisation

Simplification of TNM staging focused on consolidation of subcategories based on T staging alone; for instance, T1a, T1b, and T1c cases were aggregated under T1 [19]. This was done to focus on size as the most important factor for liver metastasis, in line with previous literature, and to strengthen the results given the small sample size [20]. Additionally, patients’ ages were grouped into six distinct categories: under 40, 40–49, 50–59, 60–69, 70–79, and 80 and above.

Tumour subtype was determined by clinical examination. Ciliochoroidal tumours were defined as those involving both the ciliary body and the choroid, while iridociliary tumours were defined as those involving the iris and the ciliary body.

### Data analyses

Statistical analysis and the generation of graphs were conducted using RStudio (2022.07.1 running R 4.2.1.) running the following packages: ‘data.table’, ‘dplyr’, ‘tidyverse’, and ‘ggplot2’ and Prism (version 9.4.1; San Diego, CA, USA). Kaplan-Meier survival analysis and Cox proportional hazards models were employed to evaluate liver metastasis and mortality rates, using the ‘survival’ package with a significance threshold of *p* < 0.05. Factors influencing liver metastasis risk were analysed according to age at treatment, TNM T category, and tumour location.

For mortality, we analysed the time from diagnosis to either the occurrence of death or the last known follow-up (censoring). For metastasis evaluation, we measured the time from diagnosis to either the first detection of liver metastasis or the most recent surveillance scan date. Median times, including time to metastasis and survival intervals, were calculated using Kaplan–Meier estimates to account for censoring. The likelihood ratio for each year was calculated by dividing the proportion of patients without metastasis in that year by the proportion of patients with metastasis in the same year. The number needed to scan (NNS) for each year was calculated as the inverse of the annual risk of developing metastasis, defined as the number of patients at risk at the start of the year divided by the number who developed metastasis during that year. For overall NNS estimates within subgroups, including T-stage and tumour location, the total number of patient-years at risk was divided by the total number of metastases within each subgroup as per study by Hagström et al. [21].

## Results

### Patients

**Fig. 1:**
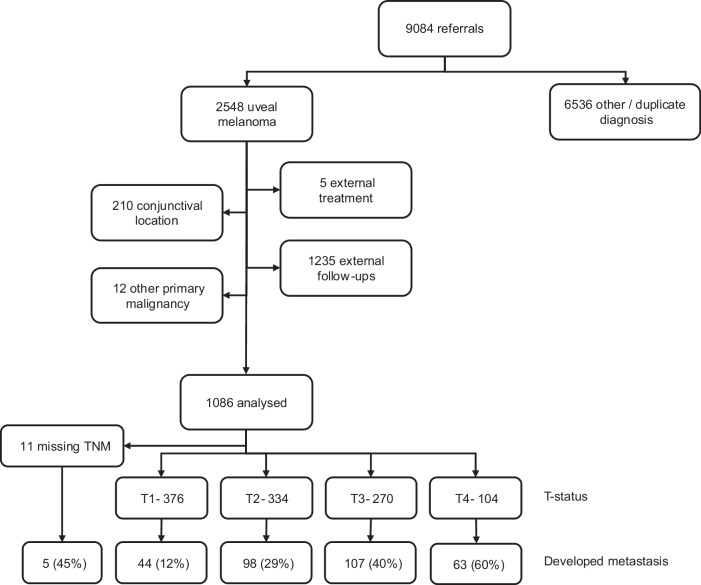
Flowchart illustrating patient selection, exclusions, and subgroup distribution for analysis.

Initially 9084 entries were recorded on our local ocular oncology database within the study timeframe. On review, 6535 entries were removed due to duplicate entries being recorded, or a diagnosis of ocular malignancy other than uveal melanoma. From these, 2548 patients with uveal melanoma were referred to our service (Fig. 1). Patients excluded from the study were as follows: 1) Patients with melanoma located in parts of the eye other than the uvea e.g. conjunctival melanoma (*n* = 210); 2) patients with either synchronous or metachronous cancer with liver metastases where there was no histological confirmation of the metastatic origin of the liver metastases (*n* = 12); 3). Patients whose surveillance imaging was performed at their local centre, where imaging reports were not retrievable or verifiable by the research team (*n* = 1235). In total 1086 patients were included and analysed. Patients (54% male and 79% White) had a median age of 68 years at diagnosis (IQR, 58–77) (Table 1). Eighty-four percent of tumours involved the choroid. The most prevalent TNM stage was T1 (35%). Globe-conserving radiation-based treatments were the most common treatment modality (overall 57%). The median length of follow-up for all patients was 3 years and 4 months (IQR 2 years and 1 month – 6 years and 8 months).

**Table 1:** Baseline clinical characteristics of included patients.

Age at diagnosis		66.9 (13.6)
Gender	Female	493 (45.4%)
Ethnicity	White	857 (79.0%)
	Asian	5 (0.5%)
	Black African	1 (0.1%)
	Any other ethnic group	3 (0.3%)
	Unknown	220 (20.1%)
T status	T1	374 (34.7%)
	T2	322 (29.9%)
	T3	267 (24.8%)
	T4	102 (9.4%)
	Data missing	11 (1.2%)
Location	Iris	18 (1.7%)
	Iridociliary	14 (1.3%)
	Ciliary body	83 (7.6%)
	Ciliochoroidal	56 (5.2%)
	Choroidal	915 (84.3%)
Treatment	Photodynamic therapy	91 (8.4%)
	Ruthenium plaque	289 (26.6%)
	Stereotactic radiosurgery	174 (16.0%)
	Proton beam	161 (14.8%)
	Local resection	7 (0.6%)
	Enucleation	346 (31.9%)
	Exenteration	6 (0.5%)
	None	11 (1.0%)
	Unknown	7 (0.6%)
Metastasis	At diagnosis	39
	Duration of study	317

### Rates of liver metastasis and survival across all patients

The Kaplan–Meier median time to liver metastasis was not reached; 25% of patients developed metastasis by 3 years 2 months, and 30% by 3 years 9 months. Among patients who developed metastasis, the crude median time from diagnosis to liver metastasis was 1 year 11 months. The Kaplan–Meier median overall survival was not reached during the follow-up period; 25% of patients had died by 4 years 3 months, and 30% by 5 years 4 months. Among patients who died, the crude median survival from diagnosis of their primary tumour was 3 years 6 months. As of July 2023, 673 (62%) patients were alive, of which 626 were disease free and 47 had liver metastases; 413 had died, of which 270 was attributed to metastatic disease, and 143 from other causes (supplementary Table 1). In total 317 patients (29%) developed a liver metastasis. Thirty-nine (prevalence of 3%) patients presented with liver metastasis at their initial surveillance scan (performed within 2 months of their diagnosis). Of these, 26 had primary tumours located in the choroid, 11 had tumours located in the ciliary body, and 2 had ciliochoroidal primaries. During the study, 317 patients developed liver metastasis. Among the 317 patients who developed liver metastasis, the Kaplan–Meier median survival interval from metastasis to death was 1 year 2 months (IQR: 6 months 3 weeks to 2 years 1 month). Among those who died, the crude median survival from metastasis to death was 11 months. With every increase in T stage, there was an associated decrease in survival time (and therefore mean surveillance period).

**Fig. 2:**
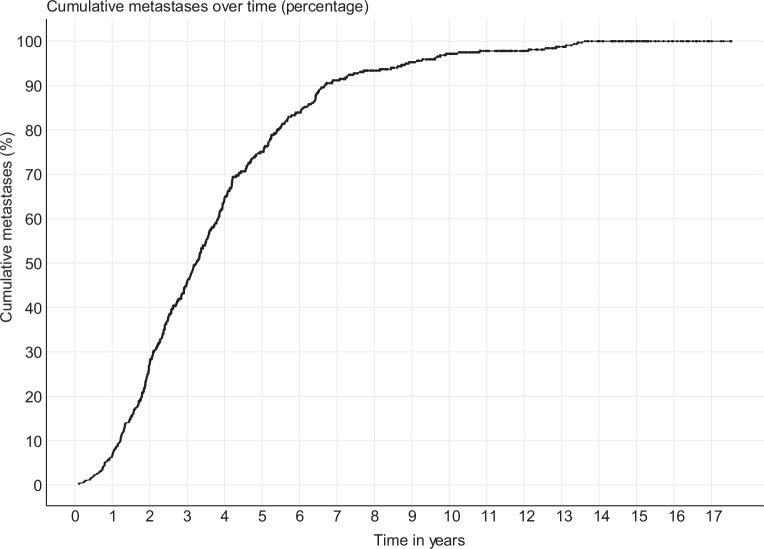
Cumulative rates of patients with metastasis across time in years.

Of those who developed liver metastasis 294 (93%) cases were detected within the first five years of surveillance (year 1, *n* = 93; year 2, *n* = 74; year 3, *n* = 56; year 4, *n* = 42; year 5, *n* = 29), with only 23 (7%) additional cases identified in the subsequent five years (Table 2 and Fig. 2). The median overall survival from diagnosis was 3 years and 3 months.

**Table 2:** Rates of liver metastasis and number needed to scan (NNS).

Years	At risk	Event (cumulative %)	Survival probability	Std error	95% CI	Likelihood ratio	NNS
1	867	93 (29.3)	0.91	0.01	0.89–0.93	1.93	12.7
2	690	74 (52.6)	0.83	0.01	0.80–0.85	1.74	11.7
3	540	56 (70.3)	0.75	0.01	0.73–0.78	1.45	12.3
4	426	42 (83.5)	0.69	0.02	0.66–0.72	1.42	12.9
5	331	29 (92.8)	0.64	0.02	0.61–0.68	1.07	14.7
6	282	7 (95)	0.63	0.02	0.59–0.66	0.40	47.3
7	214	11 (98.5)	0.60	0.02	0.56–0.64	0.47	25.6
8	172	0 (98.5)	0.60	0.02	0.56–0.64	0	U*
9	127	2 (99.1)	0.59	0.02	0.55–0.63	0.11	86.0
10	86	1 (99.4)	0.59	0.02	0.55–0.63	0.06	127.0
11	48	2 (100)	0.57	0.02	0.53–0.61	0.13	43.0

*U* Undefined as no metastases were identified in this year group.

Of the 23 patients who developed metastases more than five years after diagnosis, nine had primary tumours classified as T1, eight as T2, five as T3, and one as T4 (Table 3). Nineteen tumours originated in the choroid, two in the ciliary body, and two in the ciliochoroidal region. No patients with tumours originating in the iris, or iridociliary body developed metastases after 5 years.

**Table 3:** Kaplan–Meier–derived yearly liver metastasis events and numbers at risk stratified by T stage.

Year	T1	T2	T3	T4
1	3 (322)	21 (284)	32 (198)	35 (54)
2	9 (281)	18 (224)	34 (143)	12 (35)
3	11 (226)	17 (181)	19 (109)	7 (19)
4	8 (190)	20 (137)	10 (82)	4 (13)
5	4 (155)	14 (106)	7 (59)	4 (7)
6	4 (134)	3 (92)	0 (48)	0 (5)
7	4 (102)	0 (66)	3 (40)	1 (4)
8	0 (81)	1 (53)	0 (33)	0 (3)
9	0 (55)	1 (42)	1 (26)	0 (3)
10	0 (37)	1 (27)	0 (20)	0 (3)
11	1 (17)	0 (17)	1 (12)	0 (3)

Values are shown as the number of metastasis events (patients at risk). Patients with unknown T stage (*n* = 11) were excluded from the analysis.

### Effects of age and gender on risk of liver metastasis

Gender did not demonstrate a statistically significant impact on metastasis (Table 4). There was no clear trend across the age range, although those over 80 showed an isolated statistically significant increase in metastasis risk compared to those under 40 (HR 2.7, P 0.02).

**Table 4:** Effect of age, gender, T status and enucleation on metastasis.

		Metastasis		
Age		HR	*P*	95% CI
	<40	1.00		
	40–49	2.14	0.097	0.87– 5.26
	50–59	1.98	0.111	0.85–4.61
	60–69	2.27	0.053	0.99– 5.20
	70–79	2.10	0.077	0.92– 4.80
	80+	2.73	0.019	1.18–6.32
Gender - male		1.12	0.312	0.89–1.40
	1	1.00		
T status	2	2.80	<0.01	1.96– 3.99
	3	4.64	<0.01	3.27–6.60
	4	11.47	<0.01	7.70–16.94

### Effects of T-Status of primary tumour on risk of liver metastasis

**Fig. 3:**
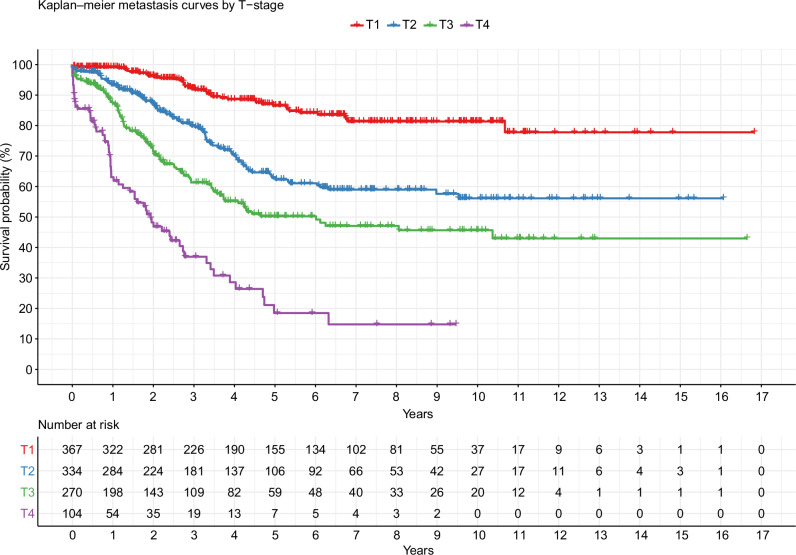
Kaplan-Meier curves of time to detect metastasis by T-status (*P* < 0.01).

This analysis included 1075 patients, excluding 11 patients due to missing TNM status. In our cohort, metastasis developed in 12% of patients with T1 disease, 29% with T2, 40% with T3, and 60% with T4 (Fig. 1). Compared to the T1 stage, there was a statistically significant (*P* < 0.01) increase in the risk of metastasis for each increase in T stage (HR 2.8 for T2, HR 4.6 for T3 and HR 11.47 for T4) (Fig. 3, Table 4).

### Effects of location of primary tumour on risk of liver metastasis

**Fig. 4:**
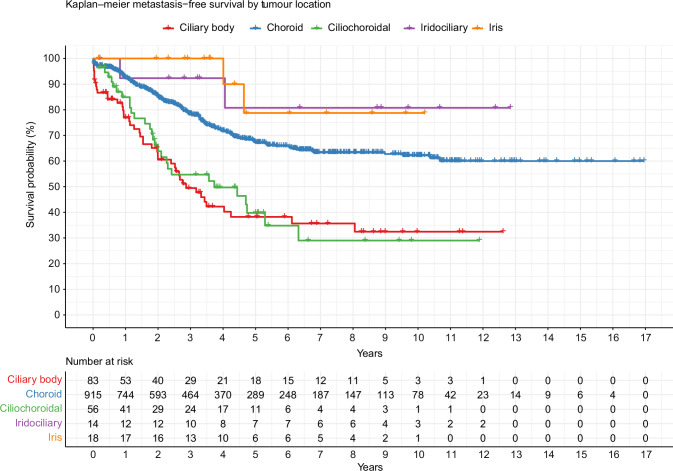
Kaplan-Meier curves of time to detect metastasis by tumour location (*P* < 0.01).

This analysis aimed to determine whether tumour location influences the risk of metastasis. As expected, the choroid was the most common site. Compared with tumours arising in the ciliary body, those located in the choroid, iridociliary region, and iris were associated with a significantly lower risk of metastasis (HR 0.38, 0.16, and 0.13 respectively; *P* < 0.01) (Table 5, Fig. 4). Although ciliochoroidal tumours also demonstrated a reduced risk, this did not reach statistical significance.

**Table 5:** Effect of tumour location on metastasis in relation to ciliary body.

Location	Metastasis (total)	Metastasis			
		HR	Std error	*P*	95% CI
Ciliary body	45 (83)	1.00			
Choroid	239 (915)	0.38	0.16	<0.01	0.27–0.52
Ciliochoroidal	29 (56)	0.89	0.24	0.64	0.56 –1.43
Iridociliary	2 (14)	0.16	0.72	<0.01	0.04–0.67
Iris	2 (18)	0.13	0.72	<0.01	0.03–0.53

In a one-vs-all location analysis, ciliary body and ciliochoroidal tumours were just over two and a half, and two times more likely to metastasise respectively (HR 2.55 and P < 0.01; HR 2.19, *P* < 0.01) (Table 6). In contrast tumours located in the choroid and iris were more favourable, being twice and more than three times less likely to metastasise, respectively, when compared with a primary tumour located anywhere else in the uvea, although the association for iris tumours did not reach statistical significance (HR 0.51, *P *< 0.01 for choroid; HR 0.29, *P* < 0.08 for iris).

**Table 6:** Effect of tumour location on metastasis in relation to other locations (grouped)

Location	Metastasis			
	HR	Std error	*P*	95% CI
Ciliary body	2.55	0.16	<0.01	1.8–3.51
Choroid	0.51	0.13	<0.01	0.40–0.66
Ciliochoroidal	2.19	0.19	<0.01	1.50–3.22
Iridociliary	0.37	0.71	0.17	0.09–1.50
Iris	0.29	0.71	0.08	0.07–1.18

### Effects of major treatments on risk of liver metastases

In our cohort 48% of patients treated with enucleation developed metastases (supplementary Table 2). Those treated with globe-conserving radiation-based treatment had a lower rate by comparison (Ruthenium plaque (17.3%), Stereotactic radiosurgery (22.4%), Proton beam therapy 28.6%), but those treated with photodynamic therapy had the lowest rate of metastasis overall (5.5%). Patients that were either treated with exenteration or received no treatment had the highest rate of metastasis of 50%, and 73% respectively.

### Number needed to scan

The 317 cases with liver metastasis resulted in an overall number needed to scan (NNS) of 15.2 to identify one patient with metastasis across all patients included in the study.

Each increase in T-stage of the primary tumour was associated with a higher rate of metastasis and a corresponding reduction in the number needed to scan (NNS): T1 = 44.3, T2 = 15.8, T3 = 9.6, and T4 = 3.9. When stratified by tumour location, the NNS was lowest for ciliary body (6.6) and ciliochoroidal tumours (6.8), with choroidal tumours at 17.3, and highest for iris (49.0) and iridociliary tumours (44.5).

When grouped by treatment type the NNS was highest for Photodynamic therapy (18.2), and lowest for those treated with exenteration (1.4). Of the four most common treatment types the NNS varied with Enucleation (2.1) being slightly lower than globe preserving radiation-based treatments (Proton beam therapy (3.5), Stereotactic radiosurgery (4.5), Ruthenium plaque (5.8)).

Additionally, whilst the NNS was relatively stable for the first five years of surveillance: year 1 (12.7), year 2 (11.7), year 3 (12.3), year 4 (12.9), year 5 (14.7) (Table 2), there was a clear change in year 6 where the NNS increased to 47.3, more than triple the previous year. Beyond this, the NNS consistently remained elevated (year 7 (25.6)), and in year 8 no metastases were identified in any surveyed patient within our study (NNS = undefined).

The NNS continued to be high for the remaining years increasing from 86 in year 9, to 127 in year 10, and culminating in a NNS of 43 in year 11. Reflecting this reduced detection yield over time, the average NNS for the first five years of surveillance was 12.8, increasing to at least 71.5 for the subsequent five years (given year 8 was undefined), an increase in the NNS by a factor of more than five and half.

## Discussion

This retrospective study aimed to determine the optimal duration of liver surveillance for detecting metastases in patients with uveal melanoma and to identify clinical factors associated with an increased risk of metastasis. We identified 1086 patients with uveal melanoma who were diagnosed in our tertiary centre between January 2006 and October 2021 who met our inclusion criteria. Despite a 15-year inclusion period, our patient population had a median duration of 3 years and 4 months of surveillance in our unit. This is similar to other studies which range from 2 years 5 months to 4 years 5 months and is most likely explained by the inherent high risk of metastases, and poor prognosis of metastatic disease heavily skewing the surveillance period [22–26]. Expectedly, with every increase in T stage, mean surveillance time decreased (supplementary Table 3). Primary tumour characteristics such as T status and location were most strongly associated with the future development of metastasis, reaching statistical significance and in keeping with previous studies [22, 27–29].

At our centre, ultrasound was almost ubiquitously preferred over MRI for routine surveillance, akin to many centres across Europe, as it offers a quicker, more available and better tolerated examination that doesn’t require intravenous contrast and can be reported on the same day as clinic appointments [22]. Currently, no comparative or controlled studies have shown a survival advantage between one method of surveillance and another [13, 16]. Our study supports this finding, showing that the median time to liver metastasis detection (in an unselected cohort) was 1 year and 11 months, comparable to Marshall et al.‘s previous study, which used regular MRI for surveillance in pre-selected high-risk patients [30]. This supports our experience that when performed by an experienced operator US is a reliable surveillance tool, and mirrors other opinions such as Balasubramanya et al. who described that, in the right hands, ultrasound can be both sensitive and specific in the evaluation of uveal melanoma metastasis, due to its ability to offer superior spatial resolution and real-time imaging [28, 30].

Our population had a slight male predominance with the majority (79%) Caucasian which is in keeping with current literature [30–33]. Notably, in 20% of cases, ethnicity was unknown, either due to a lack of self-reporting or a preference not to disclose this information. Globe-conserving radiation-based treatments were the most common treatment modality (overall 57%, which consisted of ruthenium plaque (27%), proton beam therapy (15%), stereotactic radiosurgery (16%)) with only 32% being treated with primary enucleation [25]. Whilst this widely aligns with established practice, the percentage treated with enucleation was likely marginally exacerbated in our study due its increased utility during the COVID-19 pandemic [18, 33, 34].

With reports of metastatic disease occurring up to 42 years post diagnosis, currently there is no established consensus on the optimal surveillance duration for uveal melanoma within the UK [35, 36]. However, our findings demonstrate that 93% of metastases occurred within a five-year period, with only 23 patients developing liver metastases, thereafter, leading to a quadrupling of the number needed to scan (NNS). Although data beyond 5 years is not available in most studies, Rola et al. reported that only 28% (42 patients) developed metastases more than 3 years after diagnosis, mirroring our centre’s data, where 30% (94 patients) developed metastases over the same time frame [22].

Looking beyond ocular malignancies, surveillance programmes for other cancers, such as colon cancer, accept recurrence rates of >10% after five years and do not continue systemic surveillance thereafter. Given the very low metastatic rate after 5 years in our study, it may be that reducing imaging surveillance duration to five years could be considered [37].

Whilst this reduction could ease demands on imaging resources, improve patient experience and reduce multidisciplinary team (MDT) workload, such a dramatic and sudden change to surveillance strategies may be deemed unacceptable by clinicians’ and patient groups. Therefore, a more pragmatic solution might be to consider adopting more personalised, risk-stratified surveillance strategies, mirroring those practiced in Scotland or the United States, and as advocated for in the guidelines by UK based charity Melanoma Focus [15, 16]. However, which factors are utilised to determine a patient’s risk, and therefore stratify them into a more personalised UK surveillance strategy is not necessarily clear.

Within our study, as expected, tumour size indicated by T-status was a crucial factor influencing the development of liver metastasis; with patients with T4 tumours having an 11.5-fold higher risk than those with T1 tumours. Ciliary body involvement also emerged as an important risk factor; being 8 times more likely to develop metastatic disease compared to primaries located in the iris, and more than 2.5 times more likely to metastasise than any other location, consistent with findings from previous studies[18, 29]. Whilst treatment choice was associated with different rates of metastatic disease, with Ruthenium plaque treatment having a NNS almost three times higher than enucleation, these findings could at least partly be explained by a pre-selection bias based on tumour size.

Further reflecting the influence of primary tumour factors, our results highlighted T-status and tumour location as critical factors in determining the NNS, decreasing significantly from 44 in patients with T1 tumours to 4 in patients with T4 primaries. Patients with tumours located in the iris had the highest NNS (NNS = 49), with the lowest NNS being ciliary body tumours (NNS = 6.6).

These findings align with the direction of the new Melanoma Focus UK guidelines [16], however, whilst traditionally higher T stage malignancy often leads to more intensive and perhaps longer surveillance protocols, only 1 patient with T4 disease presented with metastatic disease after 5 years (compared to 9 with T1). Therefore, in the context of more aggressive disease which is likely to metastasise early (i.e. T4 disease status), it could be argued that further screening beyond 5 years is unlikely to be beneficial for this cohort. Paradoxically, given more patients with T1/T2 disease metastasised after 5 years compared to other T stages (albeit at a lower incidence), and given the rapid progression once metastatic disease develops, this cohort could potentially yield the most benefit from prolonged screening (e.g. beyond 5 years).

Incorporating other validated prognostic markers, such as gene-expression profiling and tumour thickness may also help account for and predict the variable and often delayed onset of metastasis in patients with uveal melanoma and optimise surveillance strategies [20, 38, 39]. Metastatic disease can arise many years, or even decades, after treatment of the primary tumour, supporting the concept of a prolonged dormancy period rather than ongoing surgical dissemination as the main driver of late metastasis [40, 41]. However, in a health care system where resource use is under increasing scrutiny to provide cost-effective care, identifying which patients are likely to develop metastases is central to designing and incorporating an effective surveillance programme.

This study has several limitations. Data was collected retrospectively, from a single centre, and at a census point, rather than defined time point post diagnosis (e.g. 10 years post diagnosis). Given the rarity of uveal melanoma, a 15-year inclusion period, and grouping of T staging was also necessary to secure a robust sample size and power statistical calculations. Evolving guidelines and practices over this time frame may have also introduced potential heterogeneity into our data.

Gene expression profiling of the primary tumour was also only available in a proportion of patients, and the acquisition of new data was not covered by our ethical permissions. A dataset including this, in addition to the T stage subclassification of each tumour (e.g. T1a/b/c/d vs T1) would have undoubtedly provided additional diagnostic and prognostic information, however the subclassification of each T staging would have risked underpowering the study. Data collection also did not capture how many patients were symptomatic before the detection of metastasis, nor did it record the burden of metastatic disease.

Finally, our decision to only include data collected first hand (primary data) also significantly reduced our population group, as unretrievable local imaging reports for a large volume of patients necessitated their exclusion from the study. Whilst these limitations are not uncommon to retrospective studies, it nevertheless underscores the important role high-quality patient databases and prospective studies play, in compiling comprehensive and accurate clinical records crucial for advancing research in rare diseases.

Going forward, to help optimise surveillance strategies the creation and management of high-quality patient databases, which includes bespoke data such as primary tumour gene expression, and TNM subcategorisation is key. On a wider front, future research should also explore the health economic impact of surveillance strategies, including cost implications, effects on patient outcomes, and broader healthcare resource use, to help guide optimal pathway design.

## Conclusion

In our study, primary tumour T status and location were significant risk factors for future liver metastatic disease. Very few patients had their metastatic disease diagnosed after 5 years (7%; 23 of 331 who remained under surveillance at 5 years). This suggests that reducing the surveillance duration for uveal melanoma to five years would be in keeping with surveillance programmes for other malignancies and therefore could be considered. Further studies looking at the health economics of uveal melanoma surveillance, and combinations of risk factors would help further streamline surveillance strategies and may reinforce the potential benefits of a more efficient, personalised approach.

## Supplementary information

Supplementary tables
