# Supplementary material for: Hepatic metastasis surveillance in uveal melanoma: a retrospective cohort study from a UK tertiary centre (2006–2022)
Source: Br J Cancer. 2026 Apr 16;135(1):95–103. doi: 10.1038/s41416-026-03445-7 (PMC13270131; doi:10.1038/s41416-026-03445-7)
Supplement: Supplementary file 1 — Supplementary tables [file 41416_2026_3445_MOESM1_ESM.docx]

|  | Alive | Dead |
| --- | --- | --- |
| Metastasis | 47 | 270 |
| Disease free | 626 | 143 |
| Total | 673 | 413 |

Supplementary table 1: Distribution of alive and deceased patients according to metastatic and disease-free status.

| Treatment type | Metastasis (Overall number treated) | Percentage with metastasis | NNS |
| --- | --- | --- | --- |
| Photodynamic therapy | 5 (91) | 5.5 % | 18.2 |
| Ruthenium plaque | 50 (289) | 17.3 % | 5.8 |
| Stereotactic radiosurgery | 39 (174) | 22.4 % | 4.5 |
| Proton beam | 46 (161) | 28.6 % | 3.5 |
| Local resection | 0 (7) | U | U |
| Enucleation | 166 (346) | 48.0 % | 2.1 |
| Exenteration | 3 (6) | 50.0 % | 2 |
| None | 8 (11) | 72.7 % | 1.4 |
| Unknown | 0 (7) | U | U |

Supplementary table 2: Rates of metastatic disease and number needed to screen (NNS) based on treatment type

| T-status | Surveillance | Survival |
| --- | --- | --- |
| 1 | 4.4  (2.1-7.6) | 7.7 (4-10) |
| 2 | 3.4  (1.7-6.5) | 5.4 (3.4-9.2) |
| 3 | 2.7  (1.1-4.8) | 4.4 (2.5-7.8) |
| 4 | 1.8  (0.7-2.8) | 2.7 (1.9-4.4) |

Supplementary table 3: Median surveillance duration and survival time for each T stage, in years (lower and upper quartiles).
